# Determination of Rottlerin, a Natural Protein Kinases C Inhibitor, in Pancreatic Cancer Cells and Mouse Xenografts by RP-HPLC Method

**Published:** 2012-12-30

**Authors:** Qing-Yi Lu, Lifeng Zhang, Aurelia Lugea, Aune Moro, Mouad Edderkaoui, Guido Eibl, Stephen J. Pandol, Vay-Liang W Go

**Affiliations:** 1 Department of Medicine, David Geffen School of Medicine, University of California, Los Angeles, CA 90095, USA; 2 Veterans Affairs Greater Los Angeles Healthcare System, University of California, Los Angeles, CA, USA; 3 Department of Surgery, David Geffen School of Medicine, University of California, Los Angeles, CA 90095, USA

**Keywords:** Rottlerin, Tissue distribution, HPLC, Pancreatic cancer, *In vivo*, Cell uptake

## Abstract

Rottlerin is a natural polyphenolic ketone isolated from the pericarps of Mallotus phillippinensis. In previous studies we showed that parenteral administration of rottlerin reduced tumor growth in murine xenograft models of pancreatic cancer. The aim of this study was to develop a simple and validated method for the quantitative determination of rottlerin in plasma and tumor tissues of mice fed a rottlerin diet. A xenograft model of pancreatic cancer was prepared by injection of 2×10^6^ HPAF-II cells subcutaneously into nude mice. One week before tumor implantation, mice were randomly allocated to standard diet (AIN76A) and standard diet supplement with 0.012% rottlerin (n=6 per group). Mice were sacrificed after 6 weeks on diets. Rottlerin was extracted from the plasma and tissues using protein precipitation-extraction and analyzed by reverse-phase HPLC-DAD method. The same HPLC method was also applied to determine rottlerin levels in conditioned culture media and in cell lysates from HPAF-II cells exposed to 25 µM concentration of rottlerin. A substantial amount of rottlerin was detected in tumor (2.11 ± 0.25 nmol/g tissue) and plasma (2.88 ± 0.41 µM) in mice fed rottlerin diet. In addition, significant levels of rottlerin (57.4 ± 5.4 nmol/mg protein) were detected in cell lysates from rottlerin-treated HPAF-II cells. These data indicate that rottlerin is efficiently absorbed in cells and tissues both *in vivo* and *in vitro* and suggest a strong potential for rottlerin as a preventive or adjuvant supplement for pancreatic cancer.

## Introduction

**Figure 1:**
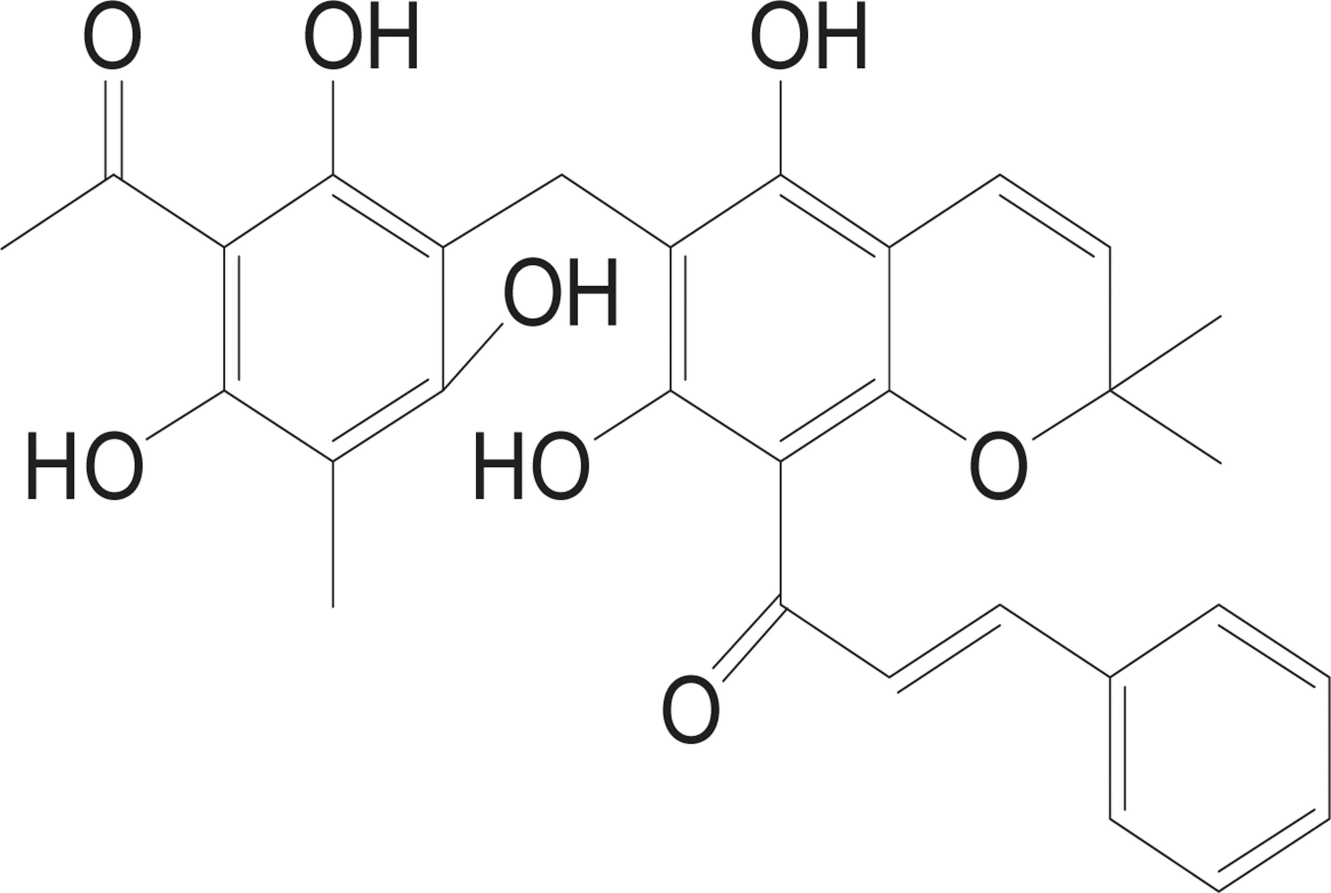
Chemical structure of rottlerin.

Rottlerin (1-[6-[(3-acetyl-2,4,6-trihydroxy-5-methylphenyl)methyl]-5,7-dihydroxy-2,2-dimethyl-2H-1-benzopyran-8yl] 3-phenyl-2-Propen-1-one, also known as Mallotoxin) is a natural polyphenolic ketone (Figure 1) isolated from the pericarps of *Mallotus phillippinensis* (common names Monkey puzzle, Monkey Face Tree, Kamala Tree). It is a traditional Indian medicine that is used against tapeworm, scabies, and herpetic ringworm. Recent scientific research has demonstrated that rottlerin has a range of molecular targets and anti-tumor activities, such as cell growth suppression [1], apoptosis [2], anti-angiogenesis [3] and inhibition of reactive oxygen species formation [4]. Rottlerin is most well-known as an inhibitor of protein kinases C (PKC) with selectivity for PKC δ [5]. It is also a mitochondrial uncoupler that depolarizes the mitochondria membrane potential, reduces cellular ATP levels and activates 5’-AMP activated protein kinase (AMPK) and affects the mitochondrial production of reactive oxygen species [6,7]. Moreover, rottlerin can target many key regulatory kinases including p38 regulated /activated kinase, cAMP-dependent protein kinase, casein kinase II, glycogen synthase, kinase 3-beta, AKT/PKB, and calmodulin-dependent kinases [8].

Our research team demonstrated recently that rottlerin at concentration range of 2.5–10 µM has potent proapoptotic and antitumor activities in pancreatic cancer *in vitro*, which is mediated by disrupting the interaction between prosurvival Bcl-2 proteins and proapoptotic BH3-only proteins [2]. Importantly, we showed rottlerin significantly reduced tumor growth in both pancreatic cancer subcutaneous and orthotopic xenograft murine models [2]. Thus rottlerin may represent a promising novel agent for the treatment of pancreatic and other cancers. However, the bioavailability of this natural compound in experimental animals has not been reported. The aims of this study were to develop a simple and validated HPLC-UV method for the quantitative determination of rottlerin in plasma, liver, pancreas and tumor tissues of mice fed with a rottlerin diet, and for the examination of rottlerin uptake by cultured pancreatic cancer cells after cells were exposed to rottlerin.

## Materials and Methods

### Reagents and chemicals

Rottlerin (96% purity), butylhydroxytoluene (BHT), ascorbic acid and β- glucuronidase/sulfatase (type H-5 from Helix Pomatia) were purchased from Sigma-Aldrich (St. Louis, MO, USA). Internal standard 3, 3′, 4′-trihydroxyflavone (97% purity) was purchased from Indofine (Hillsborough, NJ, USA). All solvents used were HPLC grade from Fisher Scientific (Pittsburg, PA, USA).

### Instrumentation

An Agilent 1100 HPLC system (Santa Clara, CA, USA) comprised of an autosampler and quaternary pump coupled to a photodiode array detector was used. The chemical separation was achieved with a RP-18 Luna column (150×4.6 mm, 3 µm) fitted with a C18 Security guard cartridge (4×3.0 mm, Phenomenex, Torrance, CA, USA). The mobile phase consisted of a binary gradient of 0.1% (v/v) ortho-phosphoric acid in water (eluent A) and acetonitrile (eluent B), used with a flow rate of 0.6 mL/min in the following conditions: 20–95% B (0–8 min); 95% B (8–19.5 min); and 95–20% B (19.5–26 min). The column temperature was held at 30°C. The chromatograms were recorded at 286 nm. Data were analyzed with the Hewlett Packard Chemstation^®^ software.

### Calibration, internal standard and quality control samples

Stock solution of rottlerin and internal standard (IS) were prepared in DMSO and ethanol, respectively, to give a final concentration of 15.5 mM (8.0 mg/mL) rottlerin and 1.0 mM IS. The stock solution of rottlerin was diluted with ethanol to obtain calibration standards at 0.12, 0.2, 0.4, 2, 5 and 20 µg/mL. IS solution was prepared by dilution of the stock solution with ethanol to a concentration of 100 µM. Quality controls samples were prepared by adding calibration solutions to the aliquots of control plasma to make rottlerin at concentrations of 200, 2000 and 8000 ng/mL. All solutions were stored at −20°C.

### Animals and diets

Animal studies were approved by the Chancellor’s Animal Research Committee of the University of California, Los Angeles, in accordance with the NIH Guide for the Care and Use of Laboratory Animals. Six week-old male athymic nude mice (Charles River Laboratories, San Diego, CA, USA) were used for the subcutaneous xenograft model of pancreatic cancer as described earlier [9]. Briefly, one week before tumor implantation, mice were randomly assigned to a control group fed with standard diet (AIN-76A, Dyets, Bethlehem, PA, USA) and to a rottlerin group fed with standard diet supplement with 0.012% w/w of rottlerin (n=6 per group). Stability of rottlerin in the diet was analyzed at day 0 (as control), 1, 2 and 3 by HPLC following vigorous extraction with ethyl acetate containing BHT as an antioxidant. Diet was replaced every 2 or 3 days based on the stability analysis. After one week on diets, each mouse was inoculated in the right flank region by subcutaneous injection with the human pancreatic tumor cell line HPAF-II (American Type Culture Collection, Manassas, VA, USA, # CRL-1997; 2×10^6^ HPAF-II cells suspended in 0.2 mL of culture medium). After tumor implantation, mice continued on diets for 5 weeks more and were then sacrificed. At sacrifice, mouse blood was taken by cardiac puncture and the tissues of interest were harvested, snap frozen in liquid nitrogen and stored at −80°C.

### Cell culture

Pancreatic cancer HPAF-II cells (2.50×10^6^) were grown in RPMI 1640 medium supplemented with 10% fetal bovine serum, 1% penicillin and streptomycin mix solution and 11.0 µg/mL sodium pyruvate (all from Invitrogen, Carlsbad, CA, USA) Cultures were maintained at 37°C in 5% CO_2_ and 95% air overnight before the addition of 25 µM of rottlerin (diluted from 50 mM stock solution in DMSO with culture medium). After incubation, cells and conditioned media were collected and analyzed as indicated in the following sections.

### Sample preparation

For the analysis of rottlerin in plasma, 10 µL of 3, 3′, 4′-trihydroxyflavone (IS, 100 µM), and 200 µL acetone containing 1% of BHT were added to 100 µL of plasma. The resulting mixture was thoroughly vortex-mixed for 2 min and then centrifuged at 1,300×g for 5 min. The precipitate was extracted one more time with acetone. Supernatants were combined and dried completely in a SpeedVac at room temperature (RT). The residue was reconstituted in 100 µL of acetone/H_2_O (80:20), and a 50 µL aliquot of the mixture was injected into the HPLC. For the analysis of tissue samples, approximately 0.15 g of frozen tissue was weighed and homogenized in 0.5 mL isotonic buffer containing 1% of ascorbic acid using a tissue grinder, which was rinsed with 0.2 mL buffer after homogenization. IS was added then mixed, followed by the addition of 1 mL acetone. The mixture was votex-mixed and then centrifuged at 5200×g for 5 min and the precipitate was extracted again with 1 mL acetone. The supernatants were combined and the solvent removed as described. The residue was reconstituted in 200 µL acetone/H_2_O (50:50) and then injected into the HPLC as described above. HPFA-II cells treated with 25 µM of rottlerin for 3 or 24 hrs. Media was collected and stored, and cells washed with ice-cold PBS three times. The dishes were placed on ice, and 100 µL of 2% ascorbic acid in water was added. After three cycles of freezing and thawing, cells were scraped, collected and sonicated. The proteins in the supernatant fraction were precipitated by adding 0.25 mL acetone and the resulting precipitate was extracted by adding the same volume of acetone. The two supernatants were combined and an aliquot was subjected to HPLC analysis. The precipitate was dried, weighed and dissolved in protein lysis buffer for protein concentration measurement using 2D Quant kit (Amersham, Piscataway, NJ, USA).

Aliquots of 100 µL cell culture medium were taken at 0 (control), 0.5, 1, 3, 6 and 24 h during cell incubation with rottlerin. Aliquots were mixed with the same volume of acetone, centrifuged and an aliquot of each supernatant was injected into the HPLC.

### Stability of rottlerin in mouse plasma and liver homogenates

An aliquot of rottlerin (20 µL, 80 µg/mL) and IS were added to 500 µL pooled blank plasma. The resulting plasma was equally divided into 5 samples. Mouse liver (0.374 g) was ground in buffer containing 1% ascorbic acid and the homogenate was equally divided into 4 samples. Rottlerin (20 µL, 16 µg/mL) was added to each sample. Samples were incubated at 37°C for 2 h and aliquots were taken at 0, 40, 80, 120 min. Rottlerin was extracted from plasma as described above. Acetone was added to liver homogenates, followed by precipitation-extraction as described above.

### Method validation

The specificity was evaluated by analyzing the chromatograms of blank plasma samples from six mice for possible interferences at the retention time of rottlerin and the IS. The analytical response was expressed as rottlerin to IS peak area ratios and calibration curves were generated by using the peak area ratios *versus* concentrations. The limit of detection (LOD) in plasma and tissues was defined as the lowest concentration resulting in a signal-to-noise ratio of 3:1. The intra-day and inter-day precision and accuracy were determined by replicative analysis of three QC samples at concentrations of 200, 2000 and 8000 ng/mL for rottlerin on the same day and on three consecutive validation days, respectively. The intra-day and inter-day precisions were expressed by the relative standard deviation (% RSD), while the relative error was used to evaluate the accuracy. The extraction recovery was determined by comparing the ratio of the analyte peak areas of the extracted QC samples with the standard solutions of the same concentration.

### Statistical analysis

Descriptive statistics, such as mean and SD, were used to summarize the results. Data were analyzed by paired student t-test. Statistical significance was defined by p-value of 0.05.

## Results and Discussion

Understanding the absorption, distribution, metabolism of a bioactive compound is important for its application as a potential chemopreventive or therapeutic agent. Extensive experimental evidence has demonstrated the correlations between tumor size and levels of the bioactive compounds found in tumor in various animal models [10,11]. To the best of our knowledge, pharmacokinetics and tissue bioavailability studies that relate efficacy and toxicity have not been carried out for rottlerin. In this paper, we describe the development of an analytical methodology which would allow the quantitative analysis of rottlerin in the mouse plasma, tissues and in pancreatic cancer cells.

Plant phenolic compounds are often found in the plasma and tissues of animals as the conjugates of glucuronide and sulfate of the parent compound. Particularly in plasma, the conjugates may be the predominant form [9,12,13]. Therefore, we first treated plasma and tissue samples by adding β-glucuronidase/sulfatase to hydrolyzed glucuronides and sulfates conjugates. After the incubation and liquid-liquid extraction, we found the samples with added β- glucuronidase/sulfatase did not produce a higher peak area by HPLC analysis in comparison to the samples without enzyme treatment, suggesting that rottlerin may not form conjugates as other phenolic compounds. Therefore, we used protein precipitation-extraction with acetone and determined the levels of rottlerin in plasma and tissues.

**Figure 2:**
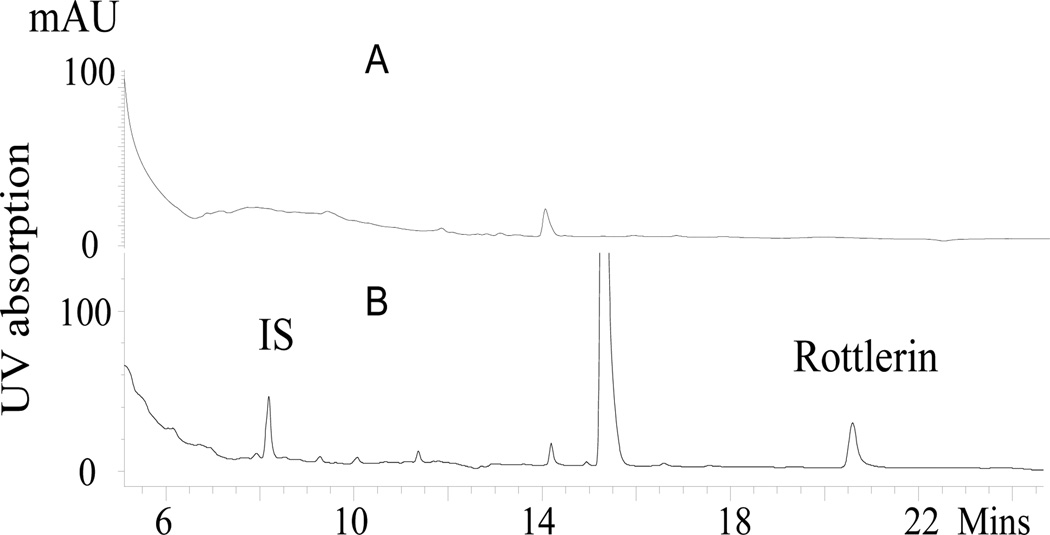
Representative HPLC chromatograms of mouse tumor monitored at 286 nm. (A) Tumor from a mouse treated with control diet (top) and (B) from rottlerin-treated mouse (bottom). The peak at 14.9 min represents BHT added as antioxidant. IS, internal standard.

Polyphenolics are known to be more stable in acidic condition. We tested the short-term (0.5, 1, 1.5 and 24 h) stability of rottlerin at pH 5.0, 7.4 and 9.4 under room temperature or 37°C. Results from the HPLC analysis showed that rottlerin, similar to the other polyphenolics, is more stable at lower pH and temperature conditions (data not shown). Water with 0.1% ortho- phosphoric acid and acetonitrile were used as HPLC mobile phase. Figure 2 illustrates a typical chromatogram of tumor tissue of rottlerin-fed mice (A) in comparison to those of control diet-fed mice (B). The HPLC profile of mouse plasma and other tissues shows a peak with same retention time at 21.7 min as standard reference rottlerin. On-line UV-VIS maxima of the diode-array detector responses facilitated the confirmation of the compound. Under the selected chromatographic condition, separation of rottlerin was achieved without the interference of endogenous peaks within the time frame of a single analysis. For the calibration curves, there were linear relationship between peak area and concentration in the range. The equation for rottlerin was y=59.614x − 0.0625 with R^2^=0.9996. The lower limit of quantitation rottlerin was 135 ng/mL.

To determine the *in vivo* effect of rottlerin on pancreatic tumor growth we used a xenograft mouse model as described in the Material and Methods section. Mice were fed standard diets supplemented with rottlerin (0.012% or 120 mg/kg diet) for 6 weeks. Based in daily food consumption, we estimated that the mice received an average of 0.5 mg/mouse/day rottlerin. Rottlerin in the diets was stable at room temperature for 3 days as determined by HPLC analysis.

The sample preparation method described above was applied to the measurement of rottlein in plasma, selected tissues, and tumors obtained from mice fed control and rottlerin diet for 6 weeks. While we determined the distribution of rottlerin in pancreas and tumor as target issues, we also investigated liver uptake as liver is a site of metastases for pancreatic cancer. Table 1 shows the average concentration of rottlerin in plasma, tumor, pancreas and liver. Levels of rottlerin were highest in liver (6.56 ± 0.83 nmol/g), followed by pancreas (2.46 ± 0.29 nmol/g) and tumor (2.11 ± 0.25 nmol/g) tissues. Average plasma levels (2.88 ± 0.41 µM) are higher than those found in liver samples. The extraction efficiency is 87.2% for plasma and 85.2–104.5% for tissues and tumors. Of note, mice fed rottlerin diet grew smaller tumors than those fed control diet (unpublished data). No rottlerin was detected in samples collected from mice fed control diet.

To examine the stability of rottlerin during the extraction procedure, we spiked rottlerin to liver tissue homogenates in pH 7.0 buffer and to plasma of control mice. The resulting samples were then incubated at 37°C for 2 h under dark condition and aliquots of each sample were taken at 40, 80, and 120 min. Table 2 lists the rottlerin concentration in each time point in comparison to the baseline. The data show that rottlerin is stable in both plasma and liver tissue homogenate under the conditions indicated.

**Figure 3:**
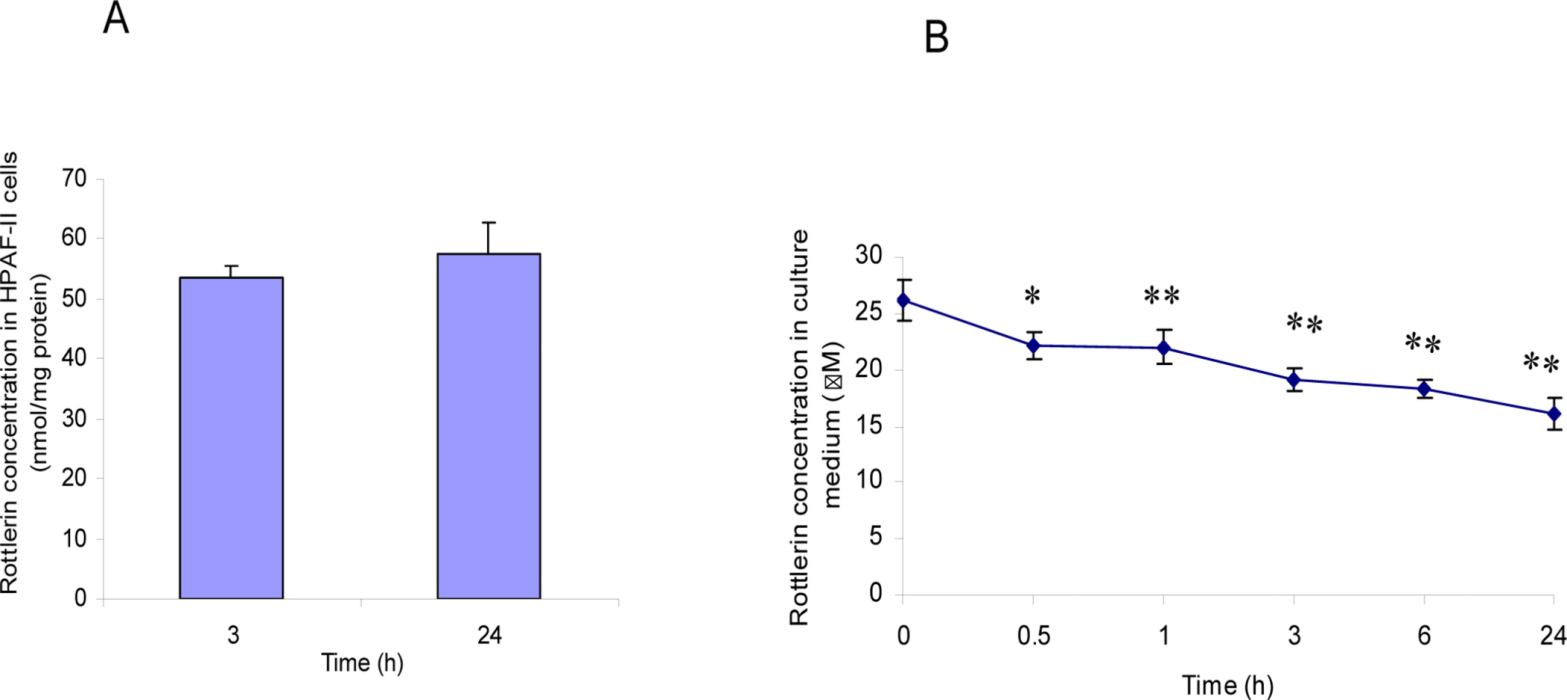
Accumulation of rottlerin in pancreatic cancer HPFA-II cells. A. Rottlerin uptake in HPFA-II cells after stimulation with 25 µM rottlerin for 3 and 24 h; and B. Change of rottlerin level in cultured media. *P<0.05, **P<0.01.

We next examined the accumulation of rottlerin in HPAF-II cells treated with 25 µM rottlerin, and the fate of rottlerin in the incubation media. Cells were collected at 3 and 24 h after rottlerin stimulation and conditioned media were taken at 0.5, 1.0, 3.0, 6.0 and 24 h (n=4 for each time point). Samples were prepared immediately for HPLC analysis without further storage. Figure 3A illustrates the absorption of rottlerin by the HPAF-II cells. In 3 h, cells accumulated 53.6 ± 1.9 nmol rottlerin per mg of total protein. At 24 h, average cellular content of rottlerin was only slightly higher (57.4 ± 5.4) than at 3 h. Therefore, based on our data, rottlerin uptake likely would plateau after 3 hrs. Of note, the peak uptake of some phenolic compounds such as quercetin and epigallocatechin-3-gallate in cancer cells have been reported at 1 or 2 h, respectively [13,14]. Figure 3B shows the time-dependant change of rottlerin concentration in the cell culture media. Rottlerin level declined from 26.25 ± 1.80 to 22.14 ± 1.26 µM in the first 30 min (a 15.7% decrease) and decreased further to 16.18 ± 1.42 µM in 24 h (a total of 38.4% decrease). This data represented a 33.3% of the rottlerin being degraded after taking into account of cellular uptake. We found similar percentage of degradation for rottlerin in another study after 24 h incubation with pH 7.4 buffer (32.9% of degradation, data not shown). Taken together, our data suggest that rottlerin is relatively stable in cell culture conditions and is readily absorbed by the HPAF-II cells.

The HPLC method was evaluated through intra-day and inter-day analysis for precision and accuracy. The accuracy and precision of the method were assessed by determining quality control (QC) samples using 4 replicated preparations of plasma samples at three concentration levels (0.2, 2 and 8 µg/mL). The accuracy of this method is 104.1, 100.9 and 100.1% at low, intermediate and high concentration, respectively. The precision indicates that all coefficients of variation (CVs) were below 10.0. Table 3 summarizes the intra-and inter-day precision and accuracy for rottlerin evaluated by assaying the quality control (QC) samples of mouse plasma as a representative example. The results demonstrated that the values were within the acceptable range and the method was accurate and precise.

To summarize, we described here a simple and a validated HPLC-UV method for the quantitative determination of rottlerin in plasma, selected tissues and tumors of the mice fed standard diet containing low content of rottlerin. Rottlerin was found in plasma, liver, pancreas and s.c. tumor tissues. Rottlerin was also found to be accumulated in pancreatic cancer HPAF-II cells and degraded gradually in culture medium with time. Our data can be used to provide important information on evaluating the potential chemo-preventive or therapeutic efficacy of rottlerin.

## Figures and Tables

**Table 1:** Rottlerin concentration in plasma and tissues of mice fed 0.012% rottlerin diet for 6 weeks (n=6).

	Rottlerin (nmol/g)
Liver	6.56 ± 0.83
Pancreas	2.46 ± 0.29
Tumor^a^	2.11 ± 0.25
Plasma (µM)	2.88 ± 0.41

**Table 2:** Stability of rottlerin in plasma and liver tissue homogenates with time (n=3).

Time (min)	Plasma (µM)	Liver tissue (nmol/g)
0	6.19 ± 0.06	6.01 ± 0.38
40	6.29 ± 0.29	5.48 ± 0.11
80	6.62 ± 0.15	5.86 ± 0.56
120	6.29 ± 0.21	5.56 ± 0.64

**Table 3:** Summary of precision and accuracy from quality control (QC) samples of mouse plasma extracts of rottlerin (n=3 days, 3 replicates per day).

Spiked (µg/ml)	Measured (µg/ml)	Accuracy (%)	Precision (%)	
			Inter-run	Intra-run
0.2	0.208	104.1	9.7	9.7
2	2.02	100.9	0.9	3.5
8	8.01	100.1	1.8	3.8
